# An Excitable Cortex and Memory Model Successfully Predicts New Pseudopod Dynamics

**DOI:** 10.1371/journal.pone.0033528

**Published:** 2012-03-22

**Authors:** Robert M. Cooper, Ned S. Wingreen, Edward C. Cox

**Affiliations:** Department of Molecular Biology, Princeton University, Princeton, New Jersey, United States of America; Université de Genève, Switzerland

## Abstract

Motile eukaryotic cells migrate with directional persistence by alternating left and right turns, even in the absence of external cues. For example, *Dictyostelium discoideum* cells crawl by extending distinct pseudopods in an alternating right-left pattern. The mechanisms underlying this zig-zag behavior, however, remain unknown. Here we propose a new Excitable Cortex and Memory (EC&M) model for understanding the alternating, zig-zag extension of pseudopods. Incorporating elements of previous models, we consider the cell cortex as an excitable system and include global inhibition of new pseudopods while a pseudopod is active. With the novel hypothesis that pseudopod activity makes the local cortex temporarily more excitable – thus creating a memory of previous pseudopod locations – the model reproduces experimentally observed zig-zag behavior. Furthermore, the EC&M model makes four new predictions concerning pseudopod dynamics. To test these predictions we develop an algorithm that detects pseudopods via hierarchical clustering of individual membrane extensions. Data from cell-tracking experiments agrees with all four predictions of the model, revealing that pseudopod placement is a non-Markovian process affected by the dynamics of previous pseudopods. The model is also compatible with known limits of chemotactic sensitivity. In addition to providing a predictive approach to studying eukaryotic cell motion, the EC&M model provides a general framework for future models, and suggests directions for new research regarding the molecular mechanisms underlying directional persistence.

## Introduction

Many eukaryotic cells move by crawling. Neutrophils migrate through the body to respond to infections. Fibroblasts crawl into and heal wounds. Metastasizing cancer cells migrate through healthy tissue to establish new tumors. *Dictyostelium discoideum* amoebae explore their environment in search of bacterial prey. At first glance these crawling cells appear to migrate randomly in the absence of external gradients, but closer inspection reveals that their motion is not that simple. Over time scales of several minutes, crawling cells maintain a relatively straight path, a phenomenon known as persistence [1], [2]. This directional persistence helps foraging amoebas or metastasizing cancer cells disperse over a larger area than they would by purely random motion; persistence helps chemotactic cells navigate up shallow or noisy gradients [3]; and persistence can help aggregating Dictyostelium maintain their direction in the waves of cAMP that propagate outward from aggregation centers [4]. Understanding directional persistence may reveal new targets for treating conditions that involve persistent cellular motion, including inflammation and metastasis.

Recent work has revealed that directional persistence arises from zig-zag motion – if a cell turns left, its next turn is more likely to be back to the right, and *vice versa* [4]. This observation was based on tracking the paths of cell centroids, and later analysis extended the observation of an alternating zig-zag pattern to time series of individual membrane extensions called pseudopods [5]. Individual pseudopods underlie the process by which many crawling cells move, including *Dictyostelium* and neutrophils (reviewed in [6]–[8]). Although individual pseudopods are less obvious in some cell types such as fibroblasts, even the broad leading edges of these cells are composed of discrete extension events [9].

Since persistence and distinct extensions are common features of crawling cell motility, a complete model of cellular motion and chemotaxis requires understanding how cells position pseudopodial extensions in a zig-zag pattern so as to maintain persistence. Despite the key role pseudopod placement plays in determining the direction of cellular motion, however, no comprehensive framework exists for describing pseudopod zig-zagging [6]–[8]. This paper presents such a framework – the excitable cortex and memory (EC&M) model – which incorporates features that are well-documented by the experimental literature to explain how cells extend pseudopods in a zig-zag fashion.

Unlike directed bacterial motility, which can be described accurately with molecular models [10], [11], regulation of eukaryotic motility is extremely complex and involves several overlapping regulatory pathways, the details of which are still being discovered [12]. Therefore, our model takes a broad top-down approach, with the aim of elucidating the general principles common to any more detailed model that shares our model's basic motif. We model pseudopods as excitable bursts from the cellular cortex, with global inhibition of new bursts as long as a pseudopod is active. When we include the key new feature of this model – a memory of previous pseudopod activity which makes that patch of cortex locally more excitable – the model reproduces the zig-zag behavior of real amoebae. Interestingly, the model also explains a previously observed increase in the likelihood of zig-zagging for pseudopods that form farther from the parent pseudopod. Moreover, the model makes four new quantitative predictions about pseudopod positioning, and we use a new pseudopod detection algorithm to test these predictions. Applying this algorithm to migrating *Dictyostelium* cells, we obtain experimental data that support all of our model's hypotheses. In addition, when the model is modified to include a gradient, simulated cells display a chemotactic sensitivity similar to that reported for live *Dictyostelium* cells [13]. Contrary to previous assumptions [14], our results show that pseudopod placement is not a Markov process depending only on whether the previous pseudopod turned left or right. Rather, new pseudopod production is a complex function of previous pseudopod placement in space and time.

## Results

### Model

The simplified EC&M model has three main features. First, pseudopods appear as bursts from an excitable cortex. Second, active pseudopods globally inhibit new pseudopod formation. Third, where a pseudopod is active it makes the cortex locally more excitable. This third feature is the key insight that leads to zig-zag behavior: previous pseudopods leave a trace of their activity, which acts as a local memory and is the basis of persistence.

The model represents the cell as a one-dimensional circle, and on this circle pseudopods stochastically appear as excitable bursts. In order to keep the model as simple as possible, we do not explicitly model excitability. Rather, we assume that excitable bursts occur along the membrane as binary events, with a rate as described below. This is a reasonable approximation given both that the times over which a pseudopod begins and ends its growth periods (each about 1 second) are much shorter on average than its total growth time (about 10 seconds), and that a pseudopod's growth rate remains fairly constant while it is growing [5]. We assume the bursting rate depends on three variables: a local memory *M*, a local inhibitor *L*, and a global inhibitor *G*. The local pseudopod production rate is given by:
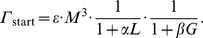
(1)The parameters *α* and *β* control the inhibitory strength of *L* and *G* respectively, and *ε* sets the overall pseudopod production rate of the system. We assume a cubic dependence of *Γ*_start_ on *M* because pseudopod activity involves much cooperative feedback [15]. The choice of such a cubic dependence is common in models of biological excitable systems, *e.g.* the FitzHugh-Nagumo model for spiking neurons [16], and the exact form of this equation is not critically important for model results.

Where a pseudopod is active we postulate that it creates a long-lived memory *M* in the cellular cortex that temporarily makes that area of the cortex more excitable:
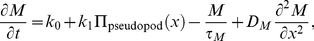
(2)where *x* represents position along the circumference of the cell and 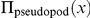 is a boxcar function that equals 1 where and when a pseudopod is active and is 0 everywhere else. The parameter *k*_1_ is the additional rate of memory production where a pseudopod is active, and to make the cortex permissive for occasional pseudopod formation everywhere, we assume that memory is formed at all locations with a low basal rate *k*_0_. We also assume the memory decays with a lifetime *τ_M_* and diffuses with coefficient *D_M_*. We chose a diffusion constant 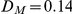 µm^2^/s, consistent with that measured for membrane-bound proteins [17].

Where a pseudopod is active it also produces a local inhibitor *L* at rate *k_L_*. This local inhibitor also decays with lifetime *τ_L_*<*τ_M_* and diffuses with diffusion constant *D_L_*:
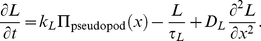
(3)The local inhibitor serves both to create a refractory period after a pseudopod stops, and to limit pseudopod lifetimes. An active pseudopod has a stopping rate that depends on the value of *L* at its center:
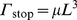
(4)where *μ* is a multiplicative coefficient. We chose a cubic dependence of *Γ*_stop_ on *L* because this form yields a distribution of pseudopod lifetimes approximating observed lifetime distributions.

Experimentally it is found that pseudopod growth suppresses formation of new pseudopods [5], so the model further assumes that when a pseudopod is active it creates a global inhibitor *G* that diffuses instantly, taking the same value at all points along the cortex. After the pseudopod stops we assume the global inhibitor instantly decays. Although we use inhibitors in this model, we note that substrate depletion could serve the same function as either the local inhibitor to limit pseudopod lifetimes and create refractory periods [18], or as the global inhibitor to suppress lateral pseudopod activity [19]. We assume instantaneous diffusion of cGMP for simplicity [20], and we note that with a diffusion constant of 300 µm^2^/s, cGMP could diffuse the length of a cell (∼10 µm) in 0.17 seconds [13], which is far faster than the pseudopod time scale.

Together, the memory, local inhibitor, and global inhibitor all determine the rate of pseudopod formation at any point along the cortex. Where the memory is high, pseudopods are more likely to begin growing, while the local and global inhibitors both suppress pseudopod formation. *M*, *L*, and *G* are dimensionless quantities with arbitrary scale. Their effects on pseudopod dynamics are set by the parameters *μ*, *ε*, *α*, and *β* in Equations 1 and 4. For further details on implementation of the model in simulations see Methods, and for parameters see Table 1.

**Table 1:** Parameters used for simulations of the EC&M model.

Parameter	Value
*k*_0_	0.1 s^−1^
*k*_1_	1.4 s^−1^
*τ_M_*	30 s
*D_M_*	0.14 µm^2^/s
*k_L_*	0.3 s^−1^
*τ_L_*	2.33 s
*D_L_*	0.1 µm^2^/s
*ε*	2.5×10^−5^ s^−1^ µm^−1^
*α*	34
*β*	11
*μ*	1 s^−1^

*k*_0_, *k*_1_, and *k_L_* are the production rates of memory *M* everywhere, the additional production rate of *M* at pseudopods, and the production rate of local inhibitor *L* at pseudopods, respectively. *τ_M_* and *τ_L_* are the lifetimes of *M* and *L*, and *D_M_* and *D_L_* are the diffusion constants of *M* and *L*. *α* and *β* set the strength of pseudopod inhibition by *L* and the global inhibitor *G*, respectively. *ε* sets the total pseudopod production rate and *μ* sets the lifetime of pseudopods.

In order to define a zig-zag, one must analyze a time series of at least three successive pseudopods, which we refer to as the grandparent, parent, and child, following Bosgraaf and van Haastert [5]. In our terminology, the parent of a new pseudopod is the most recently stopped pseudopod. If one or more pseudopod is still active when a new pseudopod starts, that which started most recently is the new pseudopod's parent. In the event that the above criteria do not define a single pseudopod, the parent is that which is spatially closest to the new pseudopod. A pseudopod's grandparent is the parent of its parent. Bosgraaf and van Haastert distinguished between “split” and “*de novo*” pseudopods based on whether or not one pseudopod appeared to grow out of a previous one, and *de novo* pseudopods were excluded from their analysis of zig-zag bias. However, both types of pseudopods are indistinguishable with respect to size and lifetime, so we include all pseudopods for analysis to avoid making *a priori* assumptions about pseudopod dynamics. In our model, pseudopods appear to split as the result of a new pseudopod beginning very close to an existing one.

We define a child pseudopod's turning angle to be the angle from the parent's location to the child's location, i.e. 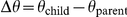. Positive values of Δ*θ* define a left turn and negative values of Δ*θ* define a right turn. We say a zig-zag has occurred when a child turns in the opposite direction of its parent, making the grandparent-parent-child series right-left or left-right. A left-left or right-right sequence would be a non-zig-zag. The zig-zag ratios reported are the number of pseudopods that are third in a zig-zag sequence divided by the number that are third in a non-zig-zag sequence.

**Figure 1:**
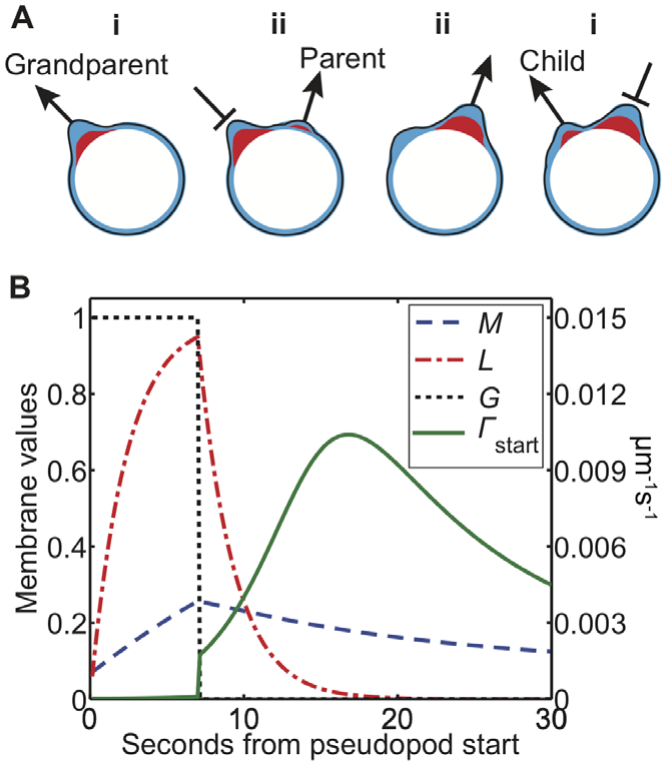
The excitable cortex and memory model. A) A sequence of three pseudopods. (i) As the first pseudopod (the grandparent) grows it generates memory (blue) and a local inhibitor (red). (ii) After some time the pseudopod stops growing and another (the parent) starts elsewhere. (iii) When the second pseudopod stops, the local inhibition at the site of the first has decayed but some memory remains. (iv) Therefore, the third pseudopod (the child) is more likely to form in the vicinity of the first, thus completing a zig-zag sequence. B) Time course of local membrane variables at the center of a simulated pseudopod. The variables shown are cortical memory *M* (dashed blue), local inhibitor *L* (dash-dotted red), global inhibitor *G* (dotted black), and pseudopod production rate *Γ*_start_ (solid green). The rate of pseudopod formation is in µm^−1^ s^−1^, while *M* and *L* are shown in units of units of 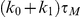 and *k_L_τ_L_*, respectively.

For an illustration of pseudopod dynamics in our model, consider the sequence of three pseudopods shown schematically in Figure 1A, with memory in blue and local inhibitor in red. In panel (i) a growing pseudopod (the grandparent) produces both cortical memory and local inhibitor. By panel (ii) the grandparent has ceased growing and its immediate vicinity is refractory due to local inhibition. A new pseudopod (the parent) begins growing to the right, and continues to grow in panel (iii). By the time the local inhibitor at the site of the grandparent has decayed, some memory remains. This memory locally increases cortical excitability, so in panel (iv), when the parent pseudopod stops growing and global inhibition lifts, a third pseudopod (the child) is most likely to form in the more excitable area left by the grandparent. This sequence of three pseudopods constitutes a zig-zag since the child turns left toward the previous location of the grandparent rather than turning right as the parent did.

Figure 1B illustrates the evolution of simulated variables at the center of a pseudopod. The rate of pseudopod formation is in units of µm^−1^ s^−1^, and *L* and *M* are in units of 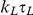 and 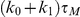, respectively. The global inhibitor *G* takes values of 0 or 1. The local inhibitor *L* rises rapidly, which increases the pseudopod's stopping rate (Eq. 4). Meanwhile the cortical memory *M* rises more slowly. When the pseudopod stops after 7 seconds, *G* decays instantly, and *L* and *M* decay according to their respective lifetimes, with *τ_L_*<*τ_M_*. The local rate of new pseudopod formation (Eq. 1) increases abruptly when *G* disappears, then the pseudopod formation rate continues to rise more slowly as *L* decays over the timescale *τ_L_*. The pseudopod production rate reaches a local peak and then declines toward its basal level as the long-lived memory *M* decays on the longer timescale *τ_M_*.

### Summary of model results and predictions

The EC&M model produces persistent random walks similar to those observed for actual cells. The model also explains the increase in likelihood of zig-zagging for pseudopods that emerge farther from their parents, a feature that was previously observed but not understood, and the model makes additional novel predictions. To test these predictions, we develop a new tracking algorithm that detects pseudopods of freely migrating *Dictyostelium* cells. Data obtained using this tracking algorithm reproduce previous observations and agrees with all four model predictions. Specifically, we find that: 1) Pseudopods are more likely to zig-zag if their parents had large turning angles. 2) Pseudopods are more likely to zig-zag if their parents did not zig-zag. 3) Pseudopods are more likely to zig-zag if their grandparents were large. 4) Pseudopods are more likely to zig-zag if they begin after a short delay from their grandparents. These results are statistically significant as tested by logistic regression, and they are described in detail below.

### The model produces a zig-zagging persistent random walk

**Figure 2:**
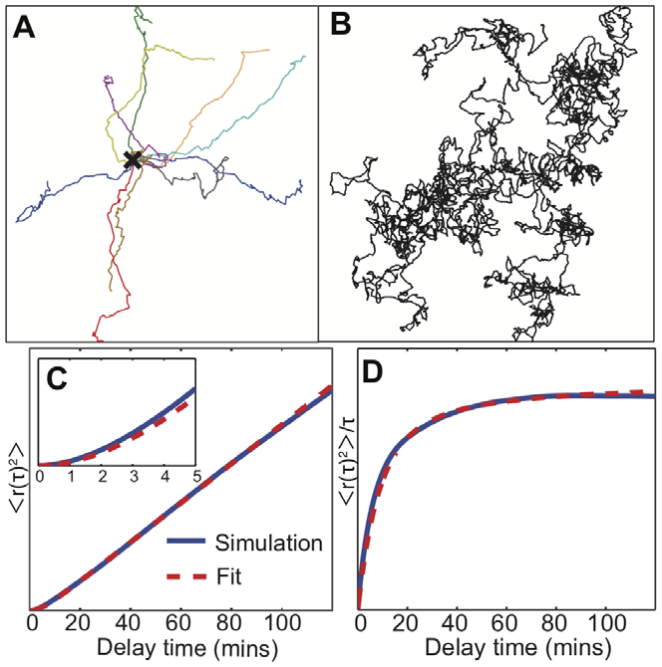
Simulated trajectories from the excitable cortex and memory (EC&M) model. A) An overlay of ten 10 minute segments of simulated paths, starting at the X in the center. The paths show persistence. The spatial scale is arbitrary and is set by the speed assigned to cells. B) A 200 hour-long simulated path. Over this much longer timescale the trajectory becomes diffusive. C) The mean squared displacement of the 200 hour path (blue), with a fit (dashed red) to the persistent random walk equation (see Methods). The persistence time from the fit is 4.0 minutes. D) The same mean squared displacement and fit plotted as 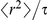 to highlight the crossover from ballistic to diffusive motion.

Figures 2A,B show sample model cell trajectories, which exhibit the same qualitative features as real cell paths. The 10 minute paths in Figure 2A show small scale zig-zag behavior and persistence, which transitions to diffusive motion over longer times as seen in the 200-hour path in Figure 2B. To quantify this crossover we plot the mean squared displacement 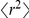 versus time lag *τ* in Figure 2C. When we fit this to the equation for a persistent random walk with constant speed *v* and persistence time *τ_p_*, 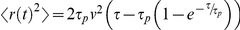 [4], the simulated trajectories exhibit a persistence time of 4.0 minutes, which is within the range of 3.4 minutes [4] and 8.8 minutes [5] reported in the literature. Figure 2D shows the mean squared displacement divided by the time lag, a quantity which is linear with positive slope for directed motion (for which 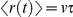), and which is constant for diffusive motion (for which 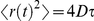). In Figure 2D one can see clearly the transition from persistent motion on short timescales to a random walk over longer timescales as 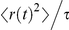 approaches a constant. As in live cells, pseudopods simulated by our model preferentially zig-zag, with a zig-zag ratio of 2.0 (*i.e.* the number of zig-zags divided by the number of non-zig-zags) for the parameters used here. Since not all pseudopods zig-zag and pseudopod angles are stochastic, over longer times, path persistence is lost.

### A new hierarchical clustering algorithm to detect pseudopods

**Figure 3:**
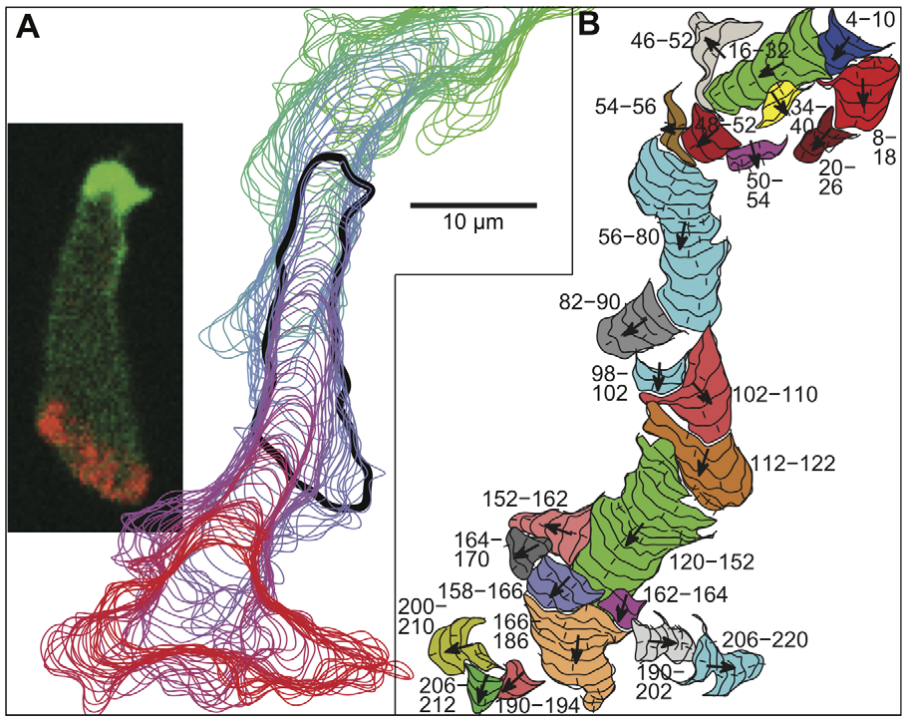
Cell tracks and pseudopods as detected by our algorithm. A) An overlay of cell contours detected in each timestep for a track covering 220 seconds. Images were captured every 2 seconds. The inset displays a fluorescent image of the cell at 120 seconds. Green is GFP-myosin, which localizes to the rear of the cell, red is RFP-LimE, which labels polymerizing actin at pseudopods, and the intensity of each channel was scaled to aid visualization. The outline in (A) corresponding to the inset image is black and in bold. B) Detected pseudopods and their component membrane extensions. Labels indicate pseudopod beginning and ending times in seconds, small hash lines show the direction of each individual extension, and arrows show the entire pseudopod's mean extension direction. See also Movie S1.

To test model results and predictions, we developed a new algorithm to track pseudopods in freely crawling cells. *Dictyostelium* cells were tagged with mRFP-LimE and GFP-Myosin to aid cell outline detection and to ensure that we reliably detected both the front and the rear of each cell (see Methods). Vegetative cells were allowed to migrate on glass coverslips under buffer with no external chemoattractant gradient, and images were captured every 2 seconds. Figure 3A shows successive cell outlines overlaid from one track (see also Movie S1). The cell was moving from top to bottom and the time goes from green to red. The inset displays the fluorescent image from this sequence at 120 seconds, and the corresponding cell outline is in bold. Cell outlines were detected using an active contour method [21], [22], and membrane extensions were found by comparing cell outlines from each time step to outlines from the previous time step (for further details, see Methods).

Pseudopods detected from these extensions are shown in Figure 3B, which has the same scale as Figure 3A. The numbers shown are the pseudopod starting and stopping times, the small hash lines indicate the angle of each individual extension, and the larger arrows show the mean extension angle of each pseudopod. To define pseudopods from individual extensions we used hierarchical clustering to group the extensions based on adjacency in time, extension angles, spatial distance, and the percentage of the newer extension which grew out of the older extension (see Methods). Consistent with the model definition, parent pseudopods were defined to be the most recently active extending pseudopod, or the most recently started if more than one pseudopod was still active, or the closest pseudopod in space if more than one was equally recent. In total we tracked 57 cells and 1764 pseudopods.

In agreement with previous results, our algorithm finds that cells extend pseudopods with a zig-zag ratio of 1.8, which is consistent with the pseudopod zig-zag ratio of 2.0 obtained from our model, and the zig-zag turning ratio of 2.1 reported by Li *et al.* [4]. Although Bosgraaf and van Haastert [5] found a higher pseudopod zig-zag ratio, near 3, our results are not directly comparable because we include all pseudopods whereas they included only pseudopod series judged to be splitting, excluding pseudopods judged to be *de novo*.

### Model predicts observed dependence of zig-zag ratio on distance from parent

**Figure 4:**
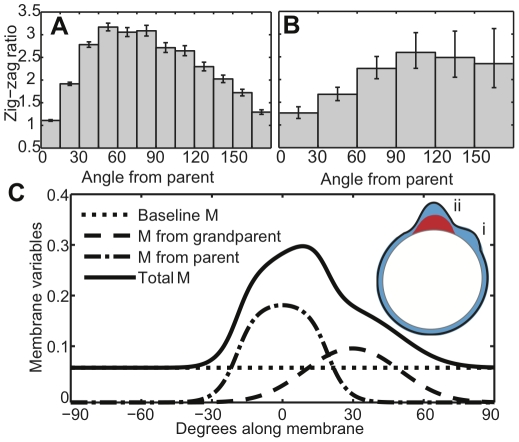
The zig-zag ratio increases with distance from the parent pseudopod. A) Simulated pseudopods are most likely to zig-zag when they extend at approximately a 90° angle from their parent. The zig-zag ratio shown is the number of left-right or right-left sequences divided by the number of left-left or right-right sequences, and error bars indicate one standard error (see Methods). B) The zig-zag ratio for pseudopods detected in tracked cells is also largest when pseudopods form nearly perpendicularly from their parent. C) Simulated membrane variables after a constructed sequence of two pseudopods (inset). The first (i) formed at 30° from 0–7 seconds, the second (ii) formed at 0° from 11–18 seconds, and the variables are shown at 18.5 seconds. Units are as in Figure 1B. The different contributions to the total cortical memory (solid blue) are shown, including baseline memory (dotted), memory from the first pseudopod (dashed), and memory from the second pseudopod (dash-dotted).

The first paper to analyze pseudopod zig-zagging found that a pseudopod forming very close to its parent was less likely to zig-zag than a pseudopod forming farther from its parent [5], although this behavior was not explained. Interestingly, simulations from our model reproduce this behavior as shown in Figure 4A, where we plot the pseudopod zig-zag ratio for pseudopods emerging at different angles from their parent. New experimental data from our pseudopod tracking algorithm (Fig. 4B) agree with both the previous observations and our model results. The zig-zag ratio in Figures 4A,B declines at 180° from the parent because of circular symmetry. Note that angular difference is directly proportional to arc-length distance for model cells, which always remain circular. Although real cells change shape as they move, their shapes remain relatively smooth and their pseudopods extend perpendicularly from the membrane [5]. Therefore a larger angular difference still generally implies a larger spatial distance along the membrane.

Figure 4C illustrates the origins of distance dependence using a constructed series of two pseudopods. The first pseudopod (i) formed at θ = 30° at time zero and lasted for 7 seconds. After an interval of 4 seconds, the second pseudopod (ii) formed at θ = 0° and lasted 7 seconds. The cortical variables are shown 0.5 seconds after the second pseudopod stopped. When the next pseudopod forms, pseudopod (i) will be its grandparent and pseudopod (ii) will be its parent.

Close to the parent (ii), the amount of local memory is high (dash-dotted blue). The memory that was generated by the grandparent (i) is lower because memory decays and diffuses over time (dashed blue). Therefore, in the area near the parent, the smaller amount of additional memory from the grandparent will make a smaller relative contribution to the total local memory (solid blue). This means that very near a parental pseudopod such as (ii) in Figure 4C, there will not be a large relative difference between the amount of memory on one side of the parent and the other, and there will not be a large inclination for a child pseudopod to zig-zag. Farther away from the parent, however, there is less total memory in the cortex. Additional memory from the grandparent would here make a larger relative contribution, increasing the probability that a child pseudopod would zig-zag. In addition, farther from the parent there is a larger spatial distance between a point on one side of the parent and a point at the same distance from the parent on the other side. If cortical excitability is a gradually varying function of distance as our model suggests, points that are farther from each other would be expected to have larger differences in excitability.

### Zig-zag ratios depend on the previous pseudopod's turn angle

Similar logic predicts that a pseudopod should be more likely to zig-zag when the parent and grandparent are farther apart (with the zig-zag ratio declining to 1 at 180° due to symmetry, as above). In the example from Figure 4C, suppose that the grandparent were very close to the parent. Then the memory and pseudopod formation rates would be nearly identical on either side of the parent, so the child would have little preference for the zig-zag side. On the other hand, consider moving the grandparent nearly opposite the parent. Then circular symmetry would make the memory similar on the parent's left and right, so the child would again have little preference to zig-zag. The zig-zag ratio of the child should reach a peak when the grandparent-parent separation is between these two extremes.

**Figure 5:**
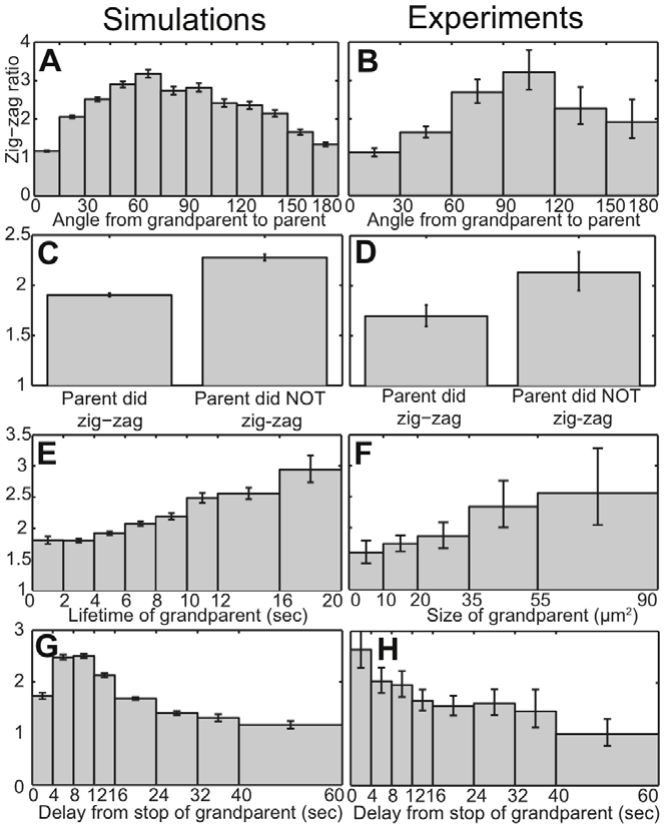
Model predictions for pseudopod zig-zag ratios (A,C,E,G) compared with experimental data (B, D, F, H). A,B) Pseudopod zig-zag ratios versus the angle from grandparent to parent. C,D) Dependence of pseudopod zig-zag ratio on whether or not the pseudopod's parent zig-zagged. E,F) Pseudopod zig-zag ratio versus the size of the grandparent pseudopod. For simulated data (E) lifetime is used as a proxy for size. G,H) Pseudopod zig-zag ratio versus the time interval from the stop of the grandparent to the start of the child.

Figure 5A shows that the simulated zig-zag ratios of child pseudopods peak when parents are approximately 90° from grandparents, as predicted by the argument above. In agreement with simulations, the experimental results with live cells show that zig-zag ratios increase as the angle from grandparent to parent increases to approximately 90° (Fig. 5B). The zig-zag ratio peaks at a larger angle in the experiments compared to the simulations, but this is likely because our simulations neglect changes in cell geometry. Cells elongate in their direction of motion, so their membrane generally has higher curvature at the leading edge. Since pseudopods extend perpendicularly to the membrane [5], pseudopods separated by a given arc length along a higher-curvature membrane region (the leading edge of a real cell) will have a larger angular difference than pseudopods separated by the same arc length along a circle with an equivalent perimeter (model cells in our simulations).

### Zig-zag ratios increase following a non-zig-zag

A simple model of pseudopod zig-zagging would suppose the choice of a left or right turn to be a Markov process, depending only on whether the previous pseudopod had turned left or right. Indeed, a previous model makes just such an assumption: every pseudopod was assigned the same probability of zig-zagging [14]. In contrast, the EC&M model predicts that zig-zagging depends on whether or not the previous pseudopod zig-zagged. Consider a non zig-zag sequence of three pseudopods, containing two consecutive turns in the same direction. On one side of the child will be two generations of memory, from both its parent and its grandparent. Any memory on the opposite side must be at least three generations old, so it will likely be close to the baseline level. Consequently, the rate of pseudopod formation will be even more biased toward the zig-zag side, *i.e.* back toward the parent and grandparent. Consistent with this logic, Figure 5C shows that model pseudopods whose parents did not zig-zag are more likely to zig-zag themselves. The experimental data in Figure 5D agree with this prediction: children of non-zig-zagging parents have a 26% higher zig-zag ratio than children of zig-zagging parents. The experimental difference between the two was significant at P<0.05 using a χ^2^ test.

### Zig-zag ratios increase following pseudopods with higher activity

In our model, cortical memory builds up as a result of pseudopod activity, so more active pseudopods should generate more memory, thereby making their grandchildren even more likely to zig-zag. Simulation results in Figure 5E illustrate this prediction, with zig-zag ratios increasing for pseudopods whose grandparents lived longer. Pseudopod lifetime is used as a proxy for pseudopod activity in the model, since all pseudopods were assumed to have the same width and activity level. With real cells, however, one can measure the size of a pseudopod to determine its total activity. The experimental results in Figure 5F reveal that grandchildren of larger pseudopods are more likely to zig-zag. The difference in zig-zag ratios between the first two bins in Figure 5F (pseudopods whose grandparents were 0–20 µm^2^) and the last two bins (pseudopods whose grandparents were 35–90 µm^2^) is significant at P<0.05 using a χ^2^ test. Figure 5F uses bins with larger ranges for larger pseudopods to reduce sampling noise, since most detected pseudopods were smaller than 20 µm^2^.

### Zig-zag ratios decrease following longer grandparent-child intervals

Not only does cortical memory build up over time in our model, but this memory also decays and diffuses with time. Thus if a child pseudopod begins growing with a long delay from the time when its grandparent ceased growing, the grandparent's memory should have dissipated and the child should be less likely to zig-zag. Figures 5G and 5H show zig-zagging dependence on time delay from the stop of a grandparent for model and real cells, respectively. In both cases, pseudopods are less likely to zig-zag with longer delays after their grandparents stopped growing. The experimental results indicate that cells lose their zig-zag bias after about 30–40 seconds, which suggests that memory diffuses and decays on a timescale of around half a minute in motile *Dictyostelium*.

### Predicted pseudopod dynamics are statistically significant in the experimental data

In agreement with model predictions, all five variables discussed above appear correlated with the probability that a pseudopod will zig-zag, including the angle of a pseudopod from its parent, the angle of the parent from the grandparent, whether or not the parent zig-zagged, the size of the grandparent, and delay time since the grandparent ceased growing (Fig. 5). To test whether the observed trends are statistically significant, we performed multiple logistic regression. The particular equation we fit was:
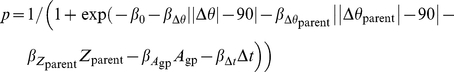
(5)where *p* is the probability of zig-zagging (note that 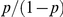 is the zig-zag ratio), Δ*θ* is the angle of a pseudopod from its parent, Δ*θ*_parent_ is the angle of the parent from the grandparent, *Z*_parent_ takes the value 1 if the parent zig-zagged and 0 otherwise, *A*_gp_ is the area of the grandparent, Δ*t* is the delay time since the grandparent stopped growing, and the corresponding *β'*s are the fit coefficients. We used the deviation of pseudopod angles from 90° because zig-zag ratios are highest at approximately 90° for both Δ*θ* (Fig. 4B) and Δ*θ*_parent_ (Fig. 5B).

The resulting best fit to experimental data shows that four of the five variables are significantly correlated with the probability of zig-zagging (Table 2). Pseudopods are less likely to zig-zag as Δ*θ* or Δ*θ*_parent_ deviates from 90° (P<10^−6^ and P<10^−10^ respectively), pseudopods are less likely to zig-zag if the parent had zig-zagged (P<0.005), and pseudopods are less likely to zig-zag as the delay time since the grandparent increases (P<0.01). Pseudopods are more likely to zig-zag as the area of their grandparent increases, but this is not a significant trend in the multiple regression analysis (P<0.14). However, when the grandparental area is used as the sole predictor in a separate logistic regression, the grandparental area does significantly predict zig-zagging (P<0.05).

**Table 2:** Multiple logistic regression results evaluating the effects of observed trends on pseudopod zig-zag probabilities.

Parameter	Value	P-value
*β*_0_	1.96	P<10^−31^
*β*_Δ*θ*_	−0.0108	P<10^−6^
*β*_Δ*θ* parent_	−0.0146	P<10^−10^
*β_Z_*_ parent_	−0.3093	P<0.005
*β_A_*_ gp_	0.0003	P<0.14
*β*_Δ*t*_	−0.0113	P<0.01

*β*_0_ is a constant offset, and the other *β'*s are fit coefficients for Δ*θ*, which is the angle of a pseudopod from its parent, Δ*θ*_parent_, which is the angle of the parent from the grandparent, *Z*_parent_, which takes the value 1 if the parent zig-zagged and 0 otherwise, *A*_gp_, which is the area of the grandparent, and Δ*t*, which is the delay time since the grandparent stopped growing. As noted in the text, *A*_gp_ is correlated with the other variables, and when it is tested in a single logistic regression, it is significant at P<0.05.

The fact that grandparental area significantly predicts zig-zagging when used as the sole predictor, but not when used in combination with the other variables in Eq. 5, suggests that the grandparental area may be correlated with some of these other variables. Indeed, the grandparental area is negatively correlated both with *Z*_parent_ (P<5×10^−4^ using a single logistic regression), and with Δ*t* (P<10^−7^ using either Kendall's or Spearman's rank correlation). When *Z*_parent_ and Δ*t* are excluded from Eq. 5, the area of the grandparental pseudopod is significant as a predictor of zig-zagging (P<0.05).

The EC&M model is consistent with these observed negative correlations between *A*_gp_ and both *Z*_parent_ and Δ*t*. The first correlation is equivalent to saying that a larger parent makes the child less likely to zig-zag. Larger parents are generally longer-lived, and longer-lived parents generally increase the time interval between grandparent and child. Since such a longer grandparent-child interval decreases the likelihood that the child will zig-zag, it follows that larger parents should have children that are less likely to zig-zag.

The second correlation – that there is a smaller grandparent-child interval when the grandparent is larger after – supports our hypothesis that pseudopod activity increases the excitability of the cellular cortex. In the framework of the EC&M model, larger grandparents create more cortical memory, which makes the cortex more excitable. This more excitable cortex then leads to shorter intervals between pseudopod bursting events. Simulation results recapitulate this finding: longer-lived grandparents are followed by children after shorter intervals.

A potential concern is that the observed trends may be influenced by difficulty in determining small turning angles. To confirm that our conclusions were not influenced by noise in extracting pseudopods with small turning angles, we excluded from analysis any pseudopods for which Δ*θ* or Δ*θ*_parent_ was less than 30°. When we performed logistic regression on this smaller dataset, we still found all variables to be significant predictors of zig-zagging (Fig. S2, again separating the grandparental area because of its correlations with the other variables).

Thus all five trends predicted by the EC&M model are statistically significant in experimental data after accounting for correlations between the trends. In addition, the observation that larger pseudopods are followed by their children and grandchildren with shorter delay times supports our hypothesis that higher pseudopod activity increases cortical excitability.

### Model accounts for chemotactic behavior

**Figure 6:**
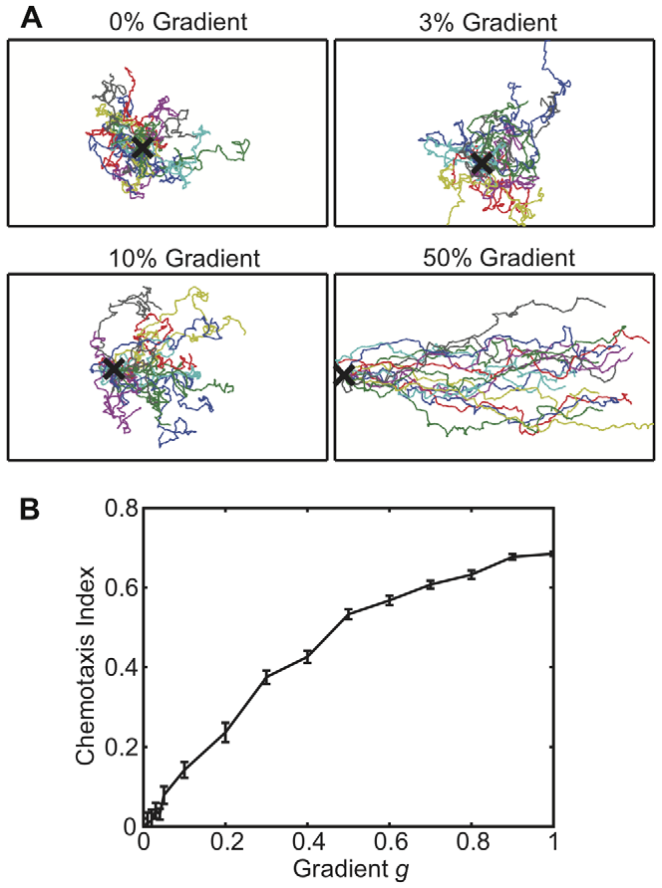
Chemotaxis simulations. A) 5 hour paths of model cells with the indicated gradient of *k*_0_. All paths are shown at the same scale, and the cells' starting point is marked with an X. B) Chemotactic index as a function of gradient, as defined in Methods. Error bars indicate the standard error of the mean over 16 simulated cells.

For many crawling cells external chemical gradients can modulate motility to produce a directed path. A chemotactic gradient creates a gradient of bound cell-surface receptors, which in turn stimulates a gradient of downstream signalling activity, which feeds into the basal motility circuit [6]–[8]. We can extend our model to include chemotaxis along external gradients by spatially varying *k*_0_, the basal production rate of *M*. Figure 6 displays typical paths and chemotactic indices from simulations in which *k*_0_ is varied, resulting in chemotactic behavior (see Methods). We find statistically significant chemotaxis in response to variation of *k*_0_ by as little as 3% across the model cell, with the chemotactic index increasing as the total variation of *k*_0_ (the “gradient”) becomes larger. Experimentally, the threshold for significant chemotaxis in *Dictyostelium* cells occurs at a receptor occupancy difference of 1% to 16% across the cell, depending on conditions [13]. Our model is consistent with this range of thresholds, even without assuming internal sharpening of gradients downstream of receptors. Importantly, model cells are still able to perform chemotaxis even when the assumed gradient in *k*_0_ is much smaller than the order of magnitude difference between *k*_0_ and *k*_1_ (see Table 1).

## Discussion

Despite recent progress, there is still no predictive framework for understanding the mechanism by which excitable membrane extension events are positioned in alternating directions to create a persistent path. Meinhardt [20] presented a polarization model that is conceptually similar to ours in having rapid local positive feedback coupled to both a slower local feedback and a weaker global feedback. However, this analysis did not include a directional memory and did not simulate cellular motion. Li et al. [4] and van Haastert [14] have presented statistical models that reproduce zig-zag behavior, but these models are not based on dynamical mechanisms, and they do not attempt to address the excitable characteristics of pseudopods. Xiong et al. [23] proposed a model for chemotaxis that incorporates pseudopod excitability, but this model does not address the zig-zag behavior or persistence of cells in the absence of a gradient. Otsuji et al. [24] presented a dynamical model that was able to produce zig-zag behavior, but which requires a potentially problematic assumption: their model includes an autocatalytic activator A that localizes to the front of the cell, an autocatalytic inhibitor B localizing to the rear, and assumes that A represses B while B activates A. We note that known biological correlates of B that localize to the rear of the cell (*e.g.* PTEN and myosin) repress pseudopod activity rather than recruiting activators [25]–[27]. Another model by Neilson et al. [28] couples a model of pseudopod dynamics to a method for evolving the cell surface to describe shape changes during cell migration. However, this model only produces pseudopods that are continuous and split symmetrically as soon as they emerge, and model cells never pause with no activity. In contrast, and consistent with previous observations [5], we observe here that real pseudopods often form away from the center of existing pseudopods, cells often follow a single pseudopod with no splitting, and cells occasionally pause with no active pseudopods (see e.g. Figure 3B). We draw upon ideas such as those discussed above and experimental evidence in the literature to propose an excitable cortex and memory (EC&M) model for understanding the excitable pseudopod dynamics that lead to persistence.

The EC&M model aims at a qualitative, high-level description to provide an intuitive framework for understanding the general principles common to the entire class of more detailed models sharing our proposed motif. For this reason, we reduce the model to three essential components: local excitable dynamics that create pseudopods, global inhibition that reduces excitability elsewhere while a pseudopod is active, and cortical memory that increases excitability at the locations of previous pseudopods. Using only these elements, the model simulates paths with persistence times within the range of those previously reported, and generates pseudopod zig-zag ratios nearly identical to those found in our tracking experiments. The model also explains the previously observed increase in zig-zag ratio for pseudopods that are farther from their parents. Furthermore, the model predicts four new features: a peak in the zig-zag ratio for a pseudopod whose parent was approximately 90° from its grandparent, a larger zig-zag ratio for children of pseudopods that did not themselves zig-zag, an increase in zig-zag ratio for pseudopods with longer-lived grandparents, and a decrease in zig-zag ratio for pseudopods that follow their grandparents after longer delays.

We test these model predictions with a new cell-tracking algorithm that detects pseudopods via hierarchical clustering of membrane extensions detected at each time step. This approach has the advantage of making very few assumptions about pseudopod dynamics, such as, for example, membrane convexity. Data from these tracking experiments agree with all of the above model predictions, and logistic regression analysis demonstrates that all observed trends are statistically significant.

The EC&M model does not attempt to describe the molecular machinery underlying pseudopod production. However, each component of the model is based on current knowledge about eukaryotic cell motility. Previous literature supports our assumptions and suggests plausible candidate molecules and mechanisms as discussed below.

### Pseudopods emerge from an excitable medium

Pseudopods and patches of pseudopod-associated proteins behave as self-organized bursts of an excitable system [23], [29], [30]. Pseudopod activity exhibits the characteristics one would expect of excitable bursts [31], including a resting state with little activity, high activity bursts with peaked lifetime distributions, a refractory period, and occasional traveling waves of pseudopod-associated proteins.

One key component of an excitable system is rapid positive feedback that creates bursts of activity. A positive feedback loop that generates pseudopods has been documented [32], [33], and this positive feedback includes many well-characterized proteins (for reviews see [6]–[8]). In addition to rapid positive feedback, an excitable system must also include slower local inhibition that both limits burst lifetimes and creates a refractory period after a burst. Consistent with such local inhibition, *Dictyostelium* pseudopods have a peaked distribution of lifetimes [5], and experimental results provide evidence of refractory periods associated with pseudopod signaling activity. For example, when latrunculin-paralyzed cells are kept in a constant chemoattractant gradient, quickly washed, and then uniformly stimulated with cAMP, the cells' previously up-gradient side is less excitable [34]. Additionally, when freely moving cells in buffer are uniformly stimulated with low levels of attractant, new signaling patches appear in younger – but not older – pseudopods, suggesting that the older pseudopods have already stopped growing and become refractory [30].

Further support for modeling pseudopods as excitable bursts comes from the observation that actin and its associated proteins travel in waves along the membrane of *Dictyostelium* cells [35]–[40], a well-known property of excitable systems with spatial diffusion. Also, when two traveling actin waves collide they mutually annihilate [35], implying that these waves leave behind a refractory zone as one would expect for waves in an excitable medium.

While pseudopod-generated local inhibition has substantial support in the literature, the underlying mechanisms are not yet clear. Several possibilities for local inhibition include recruitment of proteins inhibitory to actin polymerization such as coronin [39], [41], accumulation of inhibitory membrane lipids, substrate depletion [42], and/or physical membrane tension [43], [44].

### Global Inhibition

New pseudopods are much less likely to form while previous pseudopods are still active [5]. One key player in global inhibition appears to be cGMP [45]. cGMP is primarily produced by the sGC protein which localizes at growing pseudopods, but as a small molecule cGMP can rapidly diffuse throughout the cell, thus spreading the message that a pseudopod is active and suppressing new pseudopods elsewhere. cGMP works by promoting contractile myosin, a cortical organization incompatible with protruding actin filaments. Cells that cannot make cGMP fail to repress new pseudopods while another pseudopod is active, just as one would expect for cells lacking a global inhibitory molecule.

Another potential contributor to global inhibition is substrate depletion [19]. For example, one or several of the proteins or lipids involved in the pseudopod positive feedback loop may be available in limited quantities within the cell. As long as those pseudopod-associated proteins are sequestered in an actively growing pseudopod, there would be fewer proteins available elsewhere in the cell so the rest of the cortex would be less excitable.

A third mechanism of global inhibition is the increased cortical tension generated by protrusive activity, which was recently found to contribute to long-range pseudopod inhibition in neutrophils [46]. This mechanism allows for very rapid inhibition of new pseudopods without any delay caused by diffusive mechanisms, and it further supports our assumption of instantaneous global inhibition.

### Memory

The idea that pseudopod activity increases excitability is supported by our observation that larger pseudopods are followed by their children and grandchildren after shorter delays, and there is additional support for membrane-associated memory in the literature. For example, cells treated with latrunculin (which depolymerizes F-actin) round up and cannot move, but when these cells are exposed to chemoattractant they can still form signaling patches of pseudopod-related proteins along the membrane [30]. When these cells are exposed to a gradient of chemoattractant, the patches localize on average to the up-gradient side of the cells, but the patch formed by any individual cell is repeatably biased away from the applied gradient [47]. This directional bias has a random direction and is of variable degree in a population of cells, but for a single cell the bias is consistent over the course of ten discrete pulses, even when the direction of the gradient is changed. Such anisotropic excitability shows that cells can and do maintain a directional memory even when not moving in the direction of that bias, consistent with our hypothesis of pseudopod-generated memory.

The directional bias in latrunculin-treated cells persists for several minutes [47], but we find that zig-zag ratios in motile cells decay after approximately 30 seconds (Figure 5H). This difference in timescales suggests that an active actin cytoskeleton speeds the dynamics of cortical memory. The persistent bias of latrunculin-treated cells also suggests that when cells lack an active actin cytoskeleton, their directional memory is associated with large-scale structures that are long-lived and diffuse very slowly.

In addition to the latrunculin experiments that reveal directional memory, experiments with actively moving cells support our hypothesis that this memory is created by pseudopod activity. Uniform cAMP stimulation induces excitable patches of signaling proteins along *Dictyostelium* membranes, and new pseudopods soon grow from these patches [30], [48]. Importantly, patches are most likely to form on convex regions of the membrane. Extending pseudopods create such convex membrane domains (see e.g. Figure 3A), while membrane at the base of a pseudopod tends to be flatter or even concave. Thus, the observation that convex regions are more easily excited by cAMP is consistent with our hypothesis that recent pseudopod activity makes the membrane more locally excitable. While these examples of memory involve chemotactic stimulation, vegetative motion shares many of the same key pathways with chemotaxis [7], [8], [33], so it is reasonable to expect that directional memory is common to motion under both conditions.

Pseudopod activity alters the local membrane lipid composition, localizes many associated proteins, and changes the cortical actin meshwork. Since these modifications are produced by excitable bursts, it is reasonable to suppose that they are favorable to further excitability. In one example of persistent protein localization, when latrunculin treated cells are stimulated with attractant, at least one component of the positive feedback loop – phosphoinositide 3-kinase (PI3K) – stays localized to the membrane even after it is no longer active [32]. Such pre-localized but inactive proteins would be expected to increase local excitability. Another possibility for membrane-associated memory is large-scale cortical structure. Propagating actin waves in latrunculin-treated cells leave in their wake an altered cortical structure, which reverts to its basal state over 30–60 seconds with the help of cortexillin [49]. Contractile myosin preferentially localizes to the basal cortical state, and is excluded from the wave-produced state. Since contractile myosin and protruding actin are incompatible (reviewed in [50]), it follows that the myosin-excluding cortical structure created by actin activity should be more permissive for future actin-associated bursts than the basal cortical structure elsewhere in the cell.

Supporting a structural, cortex-based memory is the finding that the cortex at the leading edge of a migrating cell couples more weakly to the cell membrane [51]. This allows the membrane to more readily detach from the cortex and form blebs, which have been found to contribute to *Dictyostelium* motility [52]. Blebs form too rapidly to be distinguished from actin-based protrusion at our 0.5 Hz imaging rate, so the pseudopods we detect likely grow through a combination of both protrusion modes [6].

In addition to cortical memory, a recent model by Otsuji *et al.* suggests that disassembly of old pseudopods may create a localized source of molecules, which could then form a cytosolic gradient [24].

### Extensions and summary

Although the EC&M model was developed with wild-type *Dictyostelium* in mind, it could be altered very simply to describe mutants, other cell types, and chemotaxis. For example, cells lacking phospholipase A_2_ have pseudopods which grow more slowly but live longer, though the molecular mechanism is not known [5]. At first glance, one might expect longer-lived pseudopods to help *pla2*-null cells maintain a straight path longer than wild-type cells. Somewhat counterintuitively, however, despite longer-lived pseudopods these cells actually have a shorter persistence time than wild-type cells, as a result of a decreased zig-zag bias. In our model, longer lived pseudopods would give the memory of a grandparent pseudopod more time to disperse and decay, which would make grandchildren less likely to zig-zag. In this interpretation, a *pla2*-null cell would take larger steps, but each step would take so long that the cell would forget its previous direction. *gc*-null cells, which cannot produce cGMP and thus have less cortical myosin and a more excitable membrane, could be modeled by decreasing the global inhibition and increasing basal excitability. Fibroblast-like behavior can be produced by decreasing global inhibition strength so that multiple bursts occur simultaneously, though still preferentially at one region of the cell where the cortex is more excitable.

In summary, the EC&M model presented in this paper combines the two key features of excitability and zig-zagging to explain persistent cellular motion. In our model pseudopods appear as bursts of an excitable medium, and pseudopod activity makes the cortex locally more excitable, thus creating memory. Contrary to previous work, both our model and new experimental results reveal that the probability distribution of pseudopod placement depends on the details of previous pseudopod activity. The assumptions of the EC&M model are well-supported in the literature, and this model presents a new framework for interpreting future models and motivating future experiments to investigate how eukaryotic cells stay on target.

## Methods

### Simulations

Simulations were performed using MATLAB. We represented the cell as a circle with diameter 15 µm, and we discretized the model equations using a spatial resolution of *dx* = 0.1 µm and a time step of 
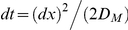
, with periodic boundary conditions. This specific time resolution was chosen to prevent numerical instability. In each timestep the variables *M* and *L* were updated according to Equations (2) and (3), and *G* was set to 1 if a pseudopod was active or 0 otherwise. Then for each active pseudopod a uniformly distributed random number was drawn from the interval 0 to 1, and if the random number was smaller than the probability of stopping at the center of the pseudopod from Equation (4), the pseudopod stopped in that timestep. Finally, at each point on the membrane the rate of pseudopod formation was found from Equation (1), another uniform random number was drawn, and if the new random number was less than the rate of formation a new pseudopod was formed at that location. All pseudopods were given the same width of 3 µm of arc length. Other parameters used for the simulation are given in Table 1.

Cell tracks were calculated by assuming cells move with constant speed in the direction of any active pseudopod. If multiple pseudopods are active the cell moves in the direction of their vector mean. To calculate the mean squared displacement versus time interval *τ*, we averaged the squared euclidean distance between all points on the path that were separated by the time interval *τ*.

To simulate external gradients we replaced *k*_0_ in Eq. 2 with
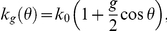
where *θ* varies from 0 to 2π around the cell, and *g* is the gradient across the cell relative to the mean, *i.e.*:
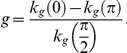
For each gradient strength, 64 cells were simulated for 5 hours each, and the chemotactic index for each cell was calculated as the distance traveled in the direction of the gradient divided by the total distance traveled, *i.e.*

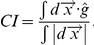



### Cell culture, microscopy, and image processing

Plasmids number 381 and 475 encoding mRFPmars-LimE [53] and GFP-myosin II [54] respectively were obtained from the dictyBase stock center (http://dictybase.org/StockCenter) [55]. *Dictyostelium discoideum* AX2 cells were transformed with these plasmids to ensure full labeling of both leading and trailing edges of cells. The transformed cells were grown on lawns of B|r-1 *E. coli* on agar plates. For microscopy, we picked vegetative cells from the feeding front, washed them of bacteria, and allowed the cells to settle in development buffer on clean glass coverslips. We imaged these cells using a confocal microscope, capturing several *z*-slices to be sure to find all extensions. We merged the two fluorescent channels, used thresholding to define cell outlines, and then refined these outlines using an active contours method [21], [22].

### Pseudopod detection

To define pseudopods, we first found individual membrane extensions in each time step by comparing the binarized cell image from each time step to the binarized image from the previous timestep. These extensions were then grouped into pseudopods using hierarchical clustering (for complete details see Methods S1 and Fig. S1). Briefly, we defined a distance metric quantifying the distance from each extension to all other extensions. This metric depends on time, spatial distance, extension angle, and the proportion of the newer extension which grew out of the older extension. Using this distance metric, the algorithm iteratively grouped the nearest two extensions, creating new grouped objects which were used in the next rounds of clustering. All clustered groups of extensions that were linked before the algorithm reached a specified distance cutoff were considered to compose a single pseudopod. Any pseudopods which did not persist for at least 2 time steps or which covered a total area less than 7.5 µm^2^ were excluded from further analysis. This hierarchical clustering approach has the advantage that one must only perform the clustering once. Then different cutoffs can be chosen according to experimental conditions so that pseudopods split where a human observer would consider them to be two distinct pseudopods. A larger distance cutoff would result in fewer splits and larger pseudopods, while a smaller distance cutoff would result in more splits and smaller pseudopods.

### Statistical analysis

The standard error of a proportion *p* of zig-zagging pseudopods out of *N* observed pseudopods is 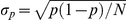. We plot zig-zag ratios 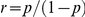 rather than proportions, so we show the standard error of the ratio as 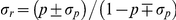. Logistic regression evaluates the importance of one or more variables in determining the probability of an event. In particular, logistic regression fits the equation 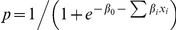, where *p* is the probability of an event, *β*_0_ is a constant offset, the *x_i_*'s are the explanatory variables, and the *β_i_*'s are fit coefficients. We performed logistic regression using the glmfit function of MATLAB, using the binomial distribution and the logit link option. Pearson and Kendall rank correlations were calculated using the corr function.

## Supporting Information

Figure S1 **Illustration of the pseudopod clustering algorithm.** A) A sample extension from a single time step. The cell was moving from left to right. A spline fit to every other pixel of the inner boundary is shown in blue and a similar spline through the outer boundary is shown in green. Dashed grey lines represent the protrusion lines, and a spline through their midpoints is shown in red. The overall angle of the extension is shown by the black arrow. B) A series of extensions, with splines through their boundaries (solid lines colored by pseudopod) and arrows showing their extension angle. The 28 extensions are numbered and labeled by the frame in which they appeared. C) A dendrogram illustrating the clustering process. Dashed lines show linkages that were formed after the distance cutoff was reached, and clusters are colored by pseudopod. D) The six pseudopods formed by the clustering algorithm. The directions of individual extensions are shown by thin lines, and the directions of pseudopods are shown as solid arrows. Solid contours mark the outer boundaries of extensions within each pseudopod.
(TIFF)

Figure S2 **Zig-zag statistics excluding pseudopods with smaller turning angles.** The group of bars on the left display statistics including all pseudopods as reported in Results, and the bars on the right display the same statistics after excluding any pseudopods for which either its own or its parent's turning angle was less than 30°. Shown within each group are the zig-zag ratio and the base-10 logarithm of the P-values as predictors of zig-zagging for the turning angle, the parent's turning angle, whether or not the parent was third in a zig-zag sequence, the area of the grandparent, and the time delay after the grandparent, as determined by a logistic regression analysis (see Methods). A separate regression was performed for *A*_gp_. The dashed line marks the cutoff for significance at P<0.05.
(TIFF)

Movie S1 **The movie corresponding to the path shown in** **Figure 3** **.** GFP-myosin is in green, localizing to the rear of the cell, and RFP-LimE is in red, localizing to the leading edge. For each frame, each channel is scaled to enhance visualization. The cell outline for the current frame is in white, and the outline for the subsequent frame is in cyan. White arrows denote extending pseudopods, and the arrows turn gray when the pseudopod stops extending. The frames are 2 seconds apart.
(MOV)

Methods S1 **Pseudopod detection via hierarchical clustering.**
(DOC)
