# Supplementary figures and images for: An Excitable Cortex and Memory Model Successfully Predicts New Pseudopod Dynamics
Source: PLoS One. 2012 Mar 22;7(3):e33528. doi: 10.1371/journal.pone.0033528 (PMC3310873; doi:10.1371/journal.pone.0033528)

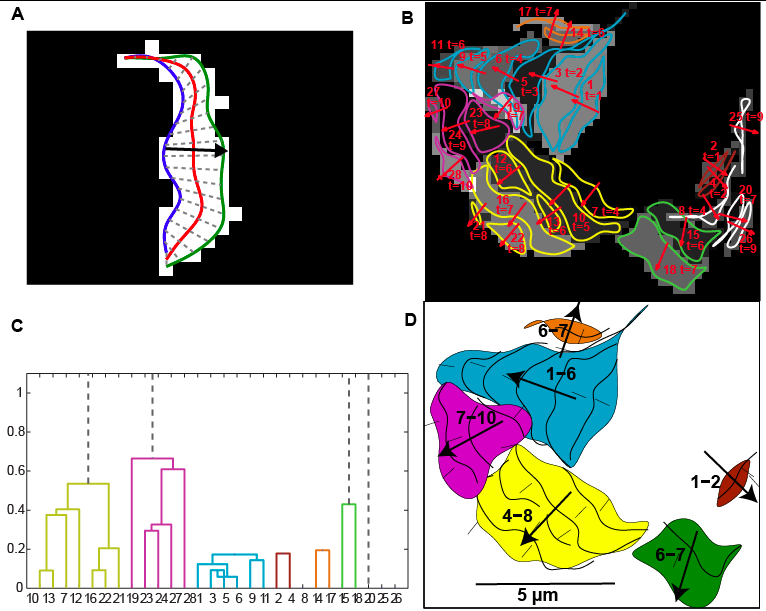

Supplement: Figure S1 — Illustration of the pseudopod clustering algorithm. A) A sample extension from a single time step. The cell was moving from left to right. A spline fit to every other pixel of the inner boundary is shown in blue and a similar spline through the outer boundary is shown in green. Dashed grey lines represent the protrusion lines, and a spline through their midpoints is shown in red. The overall angle of the extension is shown by the black arrow. B) A series of extensions, with splines through their boundaries (solid lines colored by pseudopod) and arrows showing their extension angle. The 28 extensions are numbered and labeled by the frame in which they appeared. C) A dendrogram illustrating the clustering process. Dashed lines show linkages that were formed after the distance cutoff was reached, and clusters are colored by pseudopod. D) The six pseudopods formed by the clustering algorithm. The directions of individual extensions are shown by thin lines, and the directions of pseudopods are shown as solid arrows. Solid contours mark the outer boundaries of extensions within each pseudopod. (TIFF) [file pone.0033528.s001.tiff]

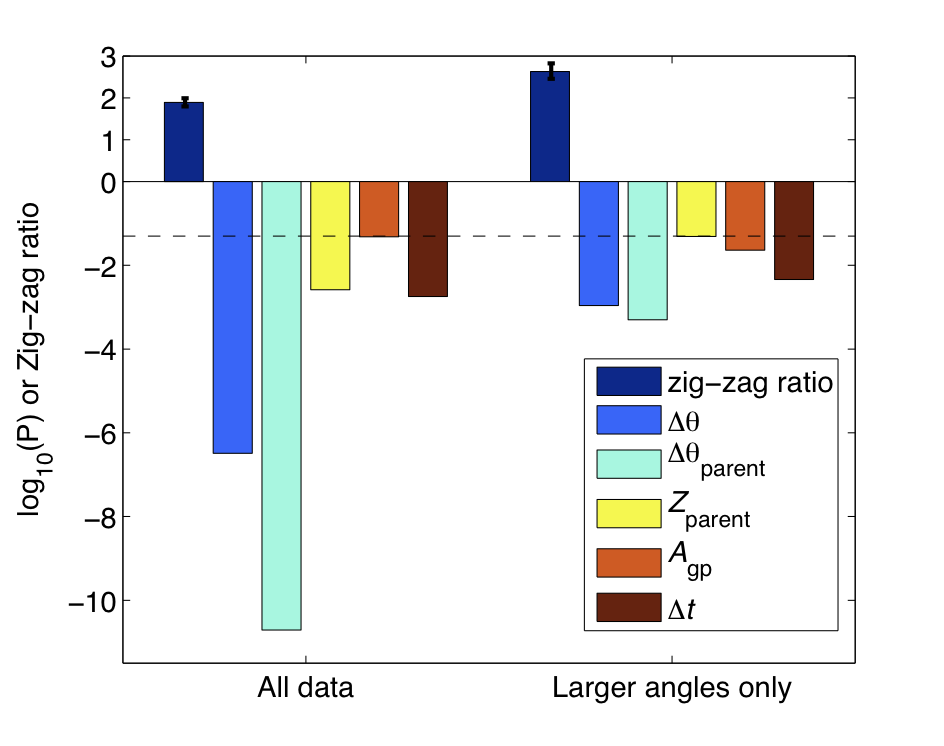

Supplement: Figure S2 — Zig-zag statistics excluding pseudopods with smaller turning angles. The group of bars on the left display statistics including all pseudopods as reported in Results, and the bars on the right display the same statistics after excluding any pseudopods for which either its own or its parent's turning angle was less than 30°. Shown within each group are the zig-zag ratio and the base-10 logarithm of the P-values as predictors of zig-zagging for the turning angle, the parent's turning angle, whether or not the parent was third in a zig-zag sequence, the area of the grandparent, and the time delay after the grandparent, as determined by a logistic regression analysis (see Methods). A separate regression was performed for A gp. The dashed line marks the cutoff for significance at P<0.05. (TIFF) [file pone.0033528.s002.tiff]
