# Supplementary material for: An Excitable Cortex and Memory Model Successfully Predicts New Pseudopod Dynamics
Source: PLoS One. 2012 Mar 22;7(3):e33528. doi: 10.1371/journal.pone.0033528 (PMC3310873; doi:10.1371/journal.pone.0033528)
Supplement: Methods S1 — Pseudopod detection via hierarchical clustering. (DOC) [file pone.0033528.s004.doc]

**Supporting Methods**

*Pseudopod detection via hierarchical clustering*

Our pseudopod detection algorithm hierarchically clusters membrane extensions into groups of extensions that extend in the same direction and are continuous in space and time. Each such group of extensions is considered to be a pseudopod. The algorithm begins with binary cell images identified as described in Methods. First, the algorithm identifies extending areas of the membrane in each time point as those pixels that are occupied by the cell in the new time point but were not occupied in the previous time point. The inner boundary of an extension (blue line in Figure S1A) is defined to be extension pixels that are adjacent to the perimeter of the cell at the previous time point. The outer boundary of an extension (green in Figure S1A) includes extension pixels that are also boundary pixels of the cell in the newer time point.

The angle of an extension is determined as illustrated in Figure S1A. First the algorithm fits splines to both the inner and outer boundaries (blue and green lines). These splines are fit through only every other pixel to produce smoother curves. Then the algorithm finds an equal number of evenly spaced points on the inner and outer splines and connects these points with “protrusion lines” (dashed grey). Next the midpoints of those protrusion lines are found, and a midline for the whole extension is formed by fitting a spline through these midpoints (red). The unit vector normal to this midline spline at its intersection with any protrusion line is defined to be the direction of that protrusion line. We define the direction of the entire extension (large black arrow) to be the vector mean of these protrusion directions, weighted by the magnitude of protrusion in that direction (the dot product of the protrusion line with its protrusion direction).

These extensions are grouped into pseudopods using hierarchical clustering, which iteratively groups the nearest two objects according to some distance metric. In our case, the grouped objects are the membrane extensions from all timesteps. Our distance metric for extensions depends on the difference between extension angles, the center-to-center spatial distance from the older extension’s outer boundary to the newer extension’s inner boundary, and the percentage of the newer extension’s inner boundary that was adjacent to the older extension’s outer boundary. For extensions not in adjacent timesteps the distance is considered infinite. The distance metric is explained more fully below.

We use the specific distance metric:

where *d*space is the spatial distance contribution, *d*angle is the angular distance contribution, and *d*adjacent is the adjacency distance contribution. Recall that Δ*t* = 2 sec means extensions were in adjacent time steps. For the spatial distance contribution we use

,

which is the euclidean distance from the center of the older extension’s outer boundary to the center of the newer extension’s inner boundary, normalized by the mean arc length of these two boundaries. The center of each boundary is weighted by the the magnitude of protrusion along its arc length. Empirically it was found that results better matched human judgment of pseudopod splitting when the spatial distance is weighted by a factor of 2. The angular distance contribution is

where Δ*θ* is the difference between the two extensions’ extension angles, and Δ*θ*max is a parameter. When , the total distance *d* will be greater than 1. The quantity is squared so that *d*angle will rise more sharply as Δ*θ* increases. For the adjacency distance contribution we use

where *ρ* is the percentage of pixels on the inner boundary of the newer extension that are adjacent to pixels on the inner boundary of the older extension, i.e. the percentage of the newer extension which grew out of the older extension. The parameter sets the minimum adjacency required for *d*adjacent to be considered 0, and the specific form was chosen so that *d*adjacent will rise sharply as the percentage of adjacent pixels decreases.

As the algorithm clusters extensions into new objects, the distance metric between these new clustered objects must be found. We define a clustered object’s angle to be the mean angle of all of its included extensions. To calculate the spatial distance and percentage of adjacent boundary for two objects, the algorithm only considers extensions that are in adjacent timesteps.

Figure S1B shows a subset of extensions from the path shown in Figure 3, which we use to demonstrate the clustering algorithm. The 28 sample extensions are labeled with a number and the frame in which they were detected (*i.e.* *t*=1-10). Splines fit to the extension boundaries are shown as solid lines, and their extension angles are shown as arrows. A dendrogram produced by the clustering algorithm is shown in Figure S1C, with clusters colored by pseudopod. Note that the metric distances do not always increase monotonically as the clustering progresses. For example, when the algorithm clustered extensions 3 and 5 together, the mean angle of the resulting object was closer to the angle of extension 6 than was the angle of extension 5 by itself, which thereby decreased the distance metric for adding extension 6 to the cluster.

To define individual pseudopods one must choose a cutoff on the distance metric for clustering. Any linkages between objects that are formed after the clustering reaches this cutoff distance are removed (dashed linkages in Figure S1C), and the objects – in this case pseudopods – are considered to be separate. We used a distance cutoff of 1, which was chosen to separate pseudopods where a human observer would typically identify two distinct pseudopods. For this example, this distance cutoff results in six distinct multi-extension pseudopods, as well as four unclustered extensions (Figure S1B-D). Any objects which did not persist for at least two timesteps or which covered an area smaller than 7.5 µm2 were excluded from analysis, thus excluding four extensions 8, 20, 25, and 26 for the example in Figure S1.
